# Multidimensional Regulatory Network and Spatiotemporal Specificity of FOXO Transcription Factors in Digestive System Tumors: Recent Advances

**DOI:** 10.3390/ijms27104191

**Published:** 2026-05-08

**Authors:** Shuo Cheng, Shidi Zhang, Jishu Quan, Ming Jin

**Affiliations:** Department of Biochemistry and Molecular Biology, Medical College, Yanbian University, Yanji 133002, China; 18093164273@163.com (S.C.); zhangshidi714@163.com (S.Z.); quanjs@ybu.edu.cn (J.Q.)

**Keywords:** digestive system tumors, FOXO family, spatiotemporal specificity, targeted therapy

## Abstract

Digestive system malignancies are a globally prevalent type of cancer that severely threatens human health. As pivotal intracellular transcriptional regulators, the forkhead box O (FOXO) protein family plays central roles in diverse physiological and pathological processes, including cell proliferation, apoptosis, and metabolism. Accumulating evidence has verified that FOXO factors exhibit dual functions as both tumor suppressors and oncoproteins. Notably, this functional duality displays a spatiotemporally specific switching pattern according to distinct stages of tumor progression and the physiological characteristics of different digestive system tumors, which poses substantial challenges for FOXO-targeted cancer therapy. Therefore, this review systematically summarizes the molecular mechanisms underlying the functional switching of FOXO in digestive system malignancies from the two dimensions of tumor progression stage and tumor-specific physiological properties, and elaborates the corresponding targeted therapeutic strategies. This work aims to provide a systematic theoretical reference for the precise intervention of digestive system tumors.

## 1. Introduction

Digestive system tumors represent a group of highly heterogeneous malignant diseases. According to the latest statistics from GLOBOCAN 2022, digestive system tumors rank among the highest in both incidence and mortality worldwide, with 4.9059 million new cases, accounting for 24.6% of all new malignant tumor cases globally. Among major subtypes, colorectal cancer has the highest incidence with 1.9264 million new cases, followed by gastric cancer (968,800 cases), liver cancer (866,100 cases), and esophageal cancer (511,100 cases), which together pose a substantial threat to human health [1]. The number of deaths caused by digestive system tumors is as high as 3.3248 million, approximately one third of all cancer related deaths worldwide. These tumors are clinically characterized by extremely low early diagnosis rate, strong invasion and metastasis ability, and significant resistance to conventional chemotherapeutic agents. Although comprehensive therapeutic strategies, including surgery, radiotherapy, chemotherapy, targeted therapy, and immunotherapy, have been continuously optimized in recent years, the overall prognosis of patients with advanced digestive system tumors remains unsatisfactory, and the 5-year postoperative recurrence rate remains persistently high. In terms of specific tumor types, the 5-year survival rate of patients with metastatic colorectal cancer is approximately 15%, while that of patients with advanced liver cancer and gastric cancer is even lower, with obvious fluctuations (mostly ranging from 10% to 20%) [1,2] due to disparities in medical care across regions. Therefore, in-depth exploration of the pathogenesis of digestive system tumors and precise identification of core regulatory targets are still critical breakthroughs for improving the diagnosis and treatment of digestive system tumors.

Within the intricate molecular networks governing cellular activities, transcription factors (TFs) participate in diverse physiological and pathological processes, including cell proliferation, apoptosis, differentiation, invasion, and metastasis by recognizing and binding to specific gene promoter regions. The FOX transcription factor superfamily has emerged as a prominent research focus in life sciences in recent years. To date, this superfamily comprises 19 distinct subfamilies (designated A–S) [3], among which the forkhead box O (FOXO) subfamily is recognized as one of the most critical regulators in cancer development and progression. In mammals, the canonical FOXO family consists of four core members: FOXO1, FOXO3, FOXO4, and FOXO6 [4], which exhibit tissue- and organ-specific expression patterns. FOXO1 and FOXO3 are ubiquitously expressed in various tissues, with particularly high abundance in the liver and pancreas. FOXO4 is significantly enriched in skeletal muscle, kidney, and colorectal tissues [5,6,7]. In contrast, FOXO6 shows a restricted distribution and is predominantly expressed in the central nervous system [8].

FOXO proteins exhibit versatile biological functions. In normal cells, FOXO acts as a tumor suppressor that maintains cellular homeostasis by enabling cells to cope with growth factor deprivation, nutrient fluctuation, and oxidative stress. In digestive system malignancies, however, FOXO displays a dual functional role: on the one hand, it retains tumor-suppressive activity; on the other hand, it frequently serves as an accomplice that allows cancer cells to evade apoptotic surveillance and initiate uncontrolled proliferation. Such functional divergence of FOXO is closely associated with distinct stages of tumor progression and different histological types of cancer [9,10,11].

In recent years, FOXO has garnered increasing attention as a therapeutic target, accompanied by a growing number of FOXO-based diagnostic and therapeutic strategies. For instance, overexpression of FOXO1 in genetically engineered CAR-T cells effectively prevents T-cell exhaustion and enhances the persistence of antitumor immunity. Meanwhile, as key regulators of T-cell homeostasis, FOXO1 and FOXO3 maintain immune tolerance in immune-active organs such as the intestine by promoting the generation of regulatory T cells (Tregs) and inhibiting the differentiation of T helper 17 (Th17) cells [12,13,14,15]. However, the dual role of FOXO as both a tumor suppressor and an oncogenic driver poses substantial challenges for its clinical translation. Accordingly, this review focuses on the molecular mechanisms underlying FOXO functional switching in digestive system tumors from the temporal dimension of tumor progression and the spatial dimension of organ-specific physiological characteristics. We provide a theoretical basis for the precise regulation of FOXO function and accelerate its clinical translation. Furthermore, we prospect the advances in precision diagnosis and personalized therapy based on FOXO regulatory patterns, aiming to offer a systematic theoretical reference for future investigations in this field.

## 2. Molecular Structure and Post-Translational Modification Regulation of the FOXO Family

The function of FOXO proteins is closely associated with their molecular structure and the sophisticated post-translational modification (PTM) system.

### 2.1. FOXO Protein Domains and Their Biological Functions

FOXO proteins contain four evolutionarily conserved functional domains with critical biological significance, and the structural integrity of these domains is a prerequisite for their transcriptional regulatory activity.

The DNA-binding domain (DBD), located at the N-terminus, determines the specific recognition of FOXO proteins for downstream target genes. The nuclear localization signal (NLS) and nuclear export signal (NES) strictly control the dynamic nucleocytoplasmic distribution of FOXO proteins. Under normal conditions, nuclear-localized FOXO proteins exhibit transcriptional activity, whereas FOXO proteins exported to the cytoplasm undergo transcriptional inactivation and even ubiquitination and degradation. The trans-activation domain (TAD) is situated at the C-terminus and is responsible for initiating the expression of target genes. Although the DBD is highly homologous among FOXO family members, sequence variations in the TAD determine their distinct binding capacities with various cofactors, thereby shaping their tissue-specific functional outputs [16,17,18,19,20].

### 2.2. Multidimensional Regulatory Network Mediated by Post-Translational Modifications

FOXO protein activity is governed by an exquisitely precise PTM system. These modifications include not only canonical phosphorylation but also acetylation, methylation, ubiquitination, SUMOylation, and other forms, which act cooperatively to endow FOXO with functional diversity in different tumors [21].

Among all modifications, phosphorylation mediated by the PI3K/Akt pathway represents the most classic and best-characterized inhibitory regulatory mechanism of FOXO in digestive system tumors. Upon stimulation by growth factors or insulin signals, activated Akt phosphorylates three conserved residues in FOXO proteins (e.g., Thr24, Ser256, and Ser391 in FOXO1; Thr32, Ser253, and Ser315 in FOXO3; Thr28, Ser193, and Ser258 in FOXO4) [4]. This modification not only masks the NLS but also generates a docking site for 14-3-3 adaptor proteins, thereby forcing FOXO to translocate out of the nucleus and be sequestered in the cytoplasm [22]. In contrast, phosphorylation induced by oxidative stress-activated c-Jun N-terminal kinase (JNK) or mediated by AMP-activated protein kinase (AMPK) exerts the opposite effect: both promote nuclear accumulation of FOXO and trigger cellular survival and defense programs [23,24]. In addition, kinases such as extracellular signal-regulated kinase (ERK) and inhibitor of nuclear factor kappa B kinase subunit beta (IKKβ) also regulate FOXO activity through phosphorylation [25,26], and this delicate balance is completely disrupted during tumorigenesis.

## 3. Time-Specific Functional Switching Mechanisms of FOXO in Digestive System Tumors

FOXO function undergoes a time-dependent switch along with tumor progression: it mainly acts as a tumor suppressor in the early stage, whereas its role converts to oncogenic when tumors advance to late-stage metastasis or drug resistance.

In early-stage esophageal cancer (EC), FOXO1 exerts a tumor-suppressive function through phosphorylation by serine/threonine kinase 3 (STK3). Phosphorylated FOXO1 shows enhanced nuclear transcriptional activity and induces the expression of tumor-suppressive genes including tumor protein P53 inducible nuclear protein 1 (TP53INP1) and cyclin-dependent kinase inhibitor 1A (CDKN1A/p21), thereby blocking malignant transformation of esophageal epithelial cells [27]. When EC progresses to the invasive stage, persistently activated oncogenic signaling pathways trigger the switch of FOXO1 from tumor-suppressive to oncogenic. In this context, FOXO1 transcriptionally activates C-C motif chemokine ligand 20 (CCL20) and colony-stimulating factor 1 (CSF-1). CCL20 increases the infiltration of peripheral blood monocytes into esophageal tissues via chemotaxis, while CSF-1 promotes the polarization of these monocytes into pro-tumor M2-type macrophages, which collectively drive distant metastasis of EC [28].

In early-stage gastric cancer (GC), FOXO1 functions as a tumor suppressor by binding to HER2 and inhibiting epithelial–mesenchymal transition (EMT) in tumor cells [29], thereby preventing tumor metastasis. When GC progresses to an advanced stage with chemoresistance, FOXO1 exhibits oncogenic properties by activating the FOXO1/glucose-6-phosphatase catalytic subunit (G6PC) axis and triggering canonical oncogenic signaling pathways such as the phosphatidylinositol 3-kinase/protein kinase B/mammalian target of rapamycin (PI3K/AKT/mTOR) pathway, ultimately enhancing resistance of tumor cells to 5-fluorouracil (5-FU) [30]. FOXO3 also acts as a tumor suppressor in early-stage GC by maintaining the transcriptional activity of intranuclear tumor-suppressive genes via dephosphorylation. When tumors progress to the late-stage metastatic phase, FOXO3 exerts oncogenic functions by directly upregulating cathepsin L (CTSL) expression, promoting extracellular matrix degradation and enhancing the migration and invasion capabilities of GC cells [31,32].

In early-stage hepatocellular carcinoma (HCC), the tumor-suppressive mechanism of FOXO3 relies on its dual modification status: dephosphorylation and acetylation. Under this modification pattern, FOXO3 cooperates with drosophila mothers against decapentaplegic protein 2/3 (Smad2/3) in the nucleus to activate the expression of pro-apoptotic genes and cell cycle arrest genes, thereby effectively inhibiting tumor proliferation [26]. In the metastatic and drug-resistant stages of advanced HCC, FOXO3 switches to an oncogenic function. Multiple clinical studies have demonstrated that FOXO3 expression is significantly higher in HCC tissues than in adjacent non-tumor tissues, and its high expression is significantly negatively correlated with overall survival (OS) [11,33]. The oncogenic mechanism of FOXO3 is attributed to its dual modification status: phosphorylation and deacetylation. In this state, FOXO3 targets Cyclin D1 and vascular endothelial growth factor (VEGF) to drive uncontrolled proliferation and angiogenesis of tumor cells [34,35]. Furthermore, FOXO3 induces protective autophagy in tumor cells, which enables cancer cells to resist the cytotoxic effects of targeted drugs such as sorafenib [36]. These findings are summarized in Table 1.

## 4. Spatial-Specific Functional Switching Mechanisms of FOXO in Digestive System Tumors

Due to the typical characteristic features of physiological functions of different organs, the mechanisms underlying the oncogenic/tumor-suppressive functional switching of FOXO vary across tissue types.

The acidic microenvironment of the gastric mucosa exerts a unique regulatory effect on the functional switching of FOXO transcription factors. SUMOylation of FOXO1 mediated by high acidity stabilizes its nuclear localization and tumor-suppressive transcriptional activity, thereby preventing abnormal proliferation of gastric cells by regulating the expression of proliferation-related genes [37,38,39]. During chronic gastric inflammation, oxidative stress elevates deSUMOylase activity and induces deSUMOylation of FOXO1. Meanwhile, the intracellular ubiquitination pathway is activated, during which representative E3 ubiquitin ligases murine double minute 2 (MDM2) or s-phase kinase-associated protein 2 (SKP2) mediate polyubiquitination of FOXO1. This modification pattern reshapes the transcriptional targeting profile of FOXO1, shifting its function from suppressing cell proliferation to activating the expression of oncogenic genes including VEGF [39,40,41,42,43]. SUMOylation and ubiquitination typically compete for the same lysine residues on FOXO1, and this modification switch represents a key mechanism underlying the functional conversion of FOXO1 during tumor progression [37].

In the fatty liver stage, FOXO1 acts as a tumor suppressor by inhibiting hepatic de novo lipogenesis and maintaining homeostasis of gluconeogenesis, thereby preventing the progression from fatty liver to HCC [44,45]. However, sustained accumulation of oxidative and nitrosative stress during this process markedly elevates intracellular reactive nitrogen species (RNS). In this specific liver microenvironment, nitrosative stress induces S-nitrosylation of cysteine residues in FOXO1, impairing its transcriptional activity related to tumor-suppressive functions. Modified FOXO1 in turn promotes the transcription of pro-tumorigenic factors such as interleukin 6 (IL-6) [46,47,48]. Overexpressed IL-6 activates the janus kinase/signal transducer and activator of transcription 3 (JAK/STAT3) signaling pathway in adjacent hepatocytes via paracrine action, forming a widespread pro-tumorigenic signaling network [48,49,50].

Bile ducts possess a unique bile acid microenvironment, with concentrations maintained within a specific physiological range. As a guardian of bile acid homeostasis, FOXO1 reduces bile acid production by binding to and repressing transcription of cytochrome P450 family 7 subfamily A member 1 (CYP7A1), a rate-limiting enzyme in bile acid synthesis, thereby preventing excessive bile acid accumulation in bile ducts [51,52,53]. When biliary obstruction or excretion dysfunction occurs, bile acid concentrations exceed the physiological threshold, representing a key driver of cholangiocarcinoma (CCA) development [51,53]. In this context, abnormally activated protein kinase C zeta (PKCζ) phosphorylates key residues of FOXO1, altering its substrate-binding preference. FOXO1 thus switches from suppressing oncogenes to directly transcriptionally activating pro-tumorigenic genes such as matrix metallopeptidase 9 (MMP9), ultimately completing its functional conversion from tumor suppressor to oncogenic driver [51,53,54,55,56].

Intestinal epithelial stem cells exhibit the highest cellular turnover rate among digestive organs, and this high-frequency proliferation meets the physiological demand for intestinal mucosal repair. To adapt to this requirement, the Wnt/β-catenin pathway is constitutively activated in intestinal epithelial stem cells, and this specific pathway status provides the basis for the functional switching of FOXO in the intestine [57,58]. Under normal physiological conditions, the sustained activation of the Wnt/β-catenin pathway in intestinal epithelial stem cells is limited and precisely regulated by FOXO4. Specifically, FOXO4 promotes the degradation of excess β-catenin by upregulating adenomatous polyposis coli 2 (APC2) [59], during which FOXO4 functions as a tumor suppressor. When abnormal activation of the pathway exceeds the physiological threshold, massively accumulated β-catenin in the nucleus reversely regulates the transcriptional activity of FOXO4 through direct binding [60]. Consequently, numerous oncogenic genes, including Cyclin D1 [61], are transcribed, marking the functional conversion of FOXO4 into an oncogenic driver. These regulatory patterns are detailed in Table 2.

## 5. Overview of Tumor-Suppressive Molecular Mechanisms of FOXO in Other Digestive System Tumors

Our previous studies have systematically elucidated the spatiotemporally specific functional switching mechanisms of the FOXO family in several digestive system tumors. This study aims to further broaden the research scope by focusing on other tumor types within the digestive system in which FOXO functional switching has not yet been documented. Summarizing the molecular mechanisms associated with FOXO function in these tumors will not only refine the functional landscape of FOXO in digestive system malignancies, but also provide a research foundation and directional reference for the subsequent exploration of potential tumor-suppressive and oncogenic switching mechanisms of FOXO in these cancers.

FOXO1 and FOXO3 function as tumor suppressors in the pancreas and salivary glands. As early-response factors, they are significantly downregulated during the precancerous lesion stage. In pancreatic and salivary gland tissues, FOXO1 and FOXO3 exert tumor-suppressive roles by regulating the expression of pro-apoptotic genes including Fas ligand (FasL) and Bcl-2-interacting mediator of cell death (Bim) [62,63]. As an early indicator of precancerous lesions, FOXO3 is markedly downregulated in pleomorphic adenomas (PAs) [64,65], while FOXO1 shows significantly reduced expression in pancreatic intraepithelial neoplasia 1 (PanIN1) lesions [63]. This responsive pattern of FOXO suggests that they serve as key regulators suppressing glandular tumorigenesis, and their loss of expression may represent a critical signal for tumor initiation.

## 6. Targeted Therapeutic Strategies of FOXO

Based on the spatiotemporally dependent mechanisms of FOXO functional switching, this review summarizes FOXO-based antitumor therapeutic strategies from both temporal and spatial dimensions. In the temporal dimension, blocking the conversion of FOXO toward an oncogenic function during the early stages of tumor development represents a key step in cancer prevention and treatment. Targeting strategies against FOXO fall into two categories, both of which have shown promising feasibility. First, the regulation of FOXO post-translational modifications. Extensive studies have systematically characterized multiple PTM mechanisms of FOXO, and key enzymes involved in these modifications, as well as their corresponding activators and inhibitors, have a well-established research and development foundation. In early-stage HCC, resveratrol and its derivatives activate silent information regulator 1 (SIRT1) to mediate deacetylation of FOXO1, thereby enhancing the expression of protective autophagy-related genes such as autophagy related 7 (Atg7) and suppressing malignant transformation [66]. In addition, inhibition of iNOS to reduce S-nitrosylation of FOXO1 can restore its tumor-suppressive function [48]. Second, there are intervention strategies targeting FOXO subcellular localization. Competitive peptides developed based on the NLS of FOXO1 (e.g., FO1-6nls) and modulators targeting the FOXO1 nuclear export receptor XPO1 (CRM1) (e.g., selinexor) interfere with the pathological nuclear exclusion of FOXO1 and forcibly retain it in the nucleus to exert tumor-suppressive effects [67,68,69]. For advanced-stage tumors, more efficient therapeutic responses can be achieved by synergistically blocking FOXO transcriptional activity and its downstream oncogenic effector axes. For example, in advanced HCC, the inhibitor AS1842856 directly binds FOXO1 and represses its transcription, effectively inhibiting the maintenance of cancer stem cell properties and angiogenesis mediated by its aberrant activation [70].

**Figure 1:**
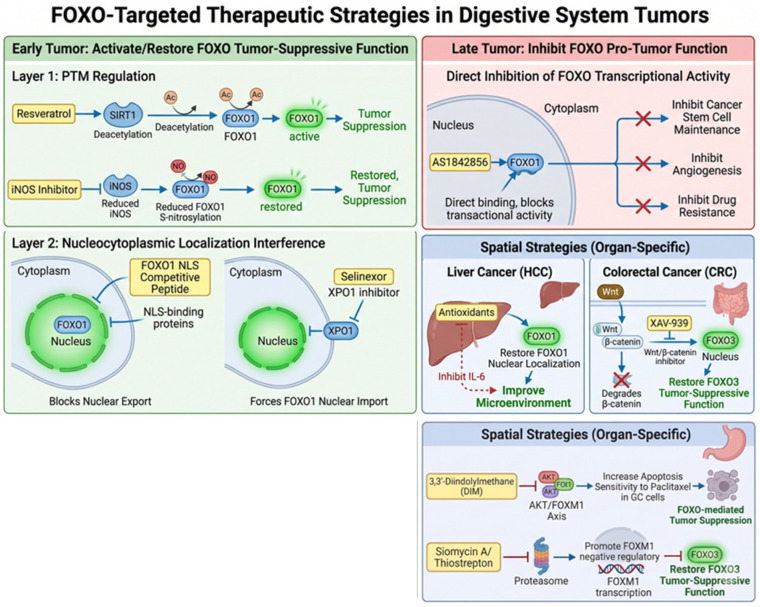
This figure illustrates the temporal and spatial targeted strategies based on the spatiotemporal functional switch of FOXO. Arrows denote promotion, whereas T-bars indicate inhibition. Temporally, early-stage tumors employ agents to activate FOXO tumor-suppressive function, while advanced tumors use inhibitors to block its oncogenic activity. Spatially, organ-specific interventions are applied in HCC and CRC to restore FOXO anti-tumor effects.

In the spatial dimension, distinct physiological characteristics of different digestive organs give rise to unique tumor microenvironments, which render the FOXO signaling axis in cancer cells organ-specific. Developing agents targeting these organ-specific FOXO signaling axes enables organ-targeted precision therapy. For instance, in HCC, given the characteristic metabolic homeostatic imbalance and oxidative stress, antioxidants can restore the nuclear localization of FOXO1, thereby suppressing abnormal IL-6 secretion and ameliorating the local inflammatory and metabolic microenvironment [48]. In colorectal cancer (CRC), the characteristically hyperactivated Wnt/β-catenin pathway promotes sustained degradation of FOXO3. Inhibition of the Wnt/β-catenin pathway using agents such as XAV-939 promotes β-catenin degradation, thus relieving the interference of β-catenin with the tumor-suppressive function of FOXO3 [71]. Targeting FOXO represents an important strategy for cancer therapy, whereas modulating the FOXO–FOXM1 antagonistic axis serves as a key complementary mechanism to enhance the tumor-suppressive activity of FOXO. FOXO3 can block the oncogenic function of FOXM1 through dual mechanisms, namely transcriptional repression and competitive promoter binding. Accordingly, inhibiting FOXM1 effectively relieves its antagonism toward FOXO3, thereby restoring and amplifying the tumor-suppressive activity of FOXO. Based on the therapeutic rationale of the FOXO–FOXM1 antagonistic axis, targeted inhibition of FOXM1 has emerged as a crucial auxiliary strategy to strengthen the tumor-suppressive function of FOXO. Accumulating evidence has verified that the natural product 3,3’-diindolylmethane (DIM) suppresses the AKT/FOXM1 signaling axis and markedly sensitizes gastric cancer cells to paclitaxel-induced apoptosis. Moreover, Siomycin A and Thiostrepton inhibit proteasome activity, promote the nuclear accumulation of negative regulators of FOXM1, block the DNA-binding and oncogenic transcriptional activity of FOXM1, and ultimately reverse FOXM1-mediated suppression of FOXO3 to reinstate its tumor-suppressive function [72,73]. The detailed regulatory network is illustrated in Figure 1.

Accumulating studies have confirmed that the oncogenic conversion of FOXO is time-dependent during tumor progression. In the future, techniques such as single-cell trajectory analysis and dynamic transcriptomics can be used to identify the temporal threshold and molecular markers of FOXO oncogenic conversion, providing a basis for early precise intervention of tumors. Meanwhile, elucidating the differences in the FOXO signaling axis among various digestive system tumors and defining the regulatory relationship between the organ-specific tumor microenvironment and FOXO activity will provide theoretical support for the development of organ-specific targeted drugs.

## Figures and Tables

**Table 1:** Temporal-specific functional switching of FOXO in digestive system tumors.

FOXO Isoform	Molecular Mechanism	Organ Type	Tumor Stage	Functional Phenotype (Oncogenic/Tumor-Suppressive)
FOXO1	Phosphorylation	Esophagus	Early stage	Tumor-Suppressive
FOXO1	Oncogenic signaling pathway-induced functional reprogramming	Esophagus	Late stage	Oncogenic
FOXO1	Binds to HER2 to inhibit EMT	Stomach	Early stage	Tumor-Suppressive
FOXO1	Activates the FOXO1/G6PC axis and stimulates the PI3K/AKT/mTOR pathway	Stomach	Late stage	Oncogenic
FOXO3	Dephosphorylation	Stomach	Early stage	Tumor-Suppressive
FOXO3	Upregulates CTSL expression	Stomach	Late stage	Oncogenic
FOXO3	Dephosphorylation + Acetylation	Liver	Early stage	Tumor-Suppressive
FOXO3	Phosphorylation + Deacetylation	Liver	Late stage	Oncogenic

**Table 2:** Spatial-specific functional switching of FOXO in digestive system tumors.

FOXO Isoform	Molecular Mechanism	Organ Type	Physiological/Pathological Characteristics of the Organ	Functional Phenotype (Tumor Suppressor/Promoter)
FOXO1	Inhibits de novo lipogenesis and maintains glucose homeostasis	Liver	Key organ for lipid and glucose metabolism (Physiological)	Tumor Suppressor
FOXO1	S-nitrosylation	Liver	Oxidative stress (Pathological)	Tumor Promoter
FOXO4	Upregulates APC2 to promote β-catenin degradation	Intestine	Moderate activation of Wnt/β-catenin signaling (Physiological)	Tumor Suppressor
FOXO4	Binds to accumulated β-catenin in the nucleus and transcriptionally activates Cyclin D1	Intestine	Excessive activation of Wnt/β-catenin signaling (Pathological)	Tumor Promoter
FOXO1	SUMOylation	Stomach	Acidic microenvironment (Physiological)	Tumor Suppressor
FOXO1	DeSUMOylation plus ubiquitination modification	Stomach	Chronic inflammation (Pathological)	Tumor Promoter
FOXO1	Inhibits CYP7A1 transcription to maintain bile acid homeostasis	Bile Duct	Bile acid concentration within physiological threshold (Physiological)	Tumor Suppressor
FOXO1	Phosphorylation	Bile Duct	Biliary obstruction and excessive accumulation of bile acids (Pathological)	Tumor Promoter

## Data Availability

No new data were created or analyzed in this study. Data sharing is not applicable to this article.

## Abbreviations

The following abbreviations are used in this manuscript:

FOXO	Forkhead Box O
PTM	Post-Translational Modification
EC	Esophageal Cancer
GC	Gastric Cancer
HCC	Hepatocellular Carcinoma
CRC	Colorectal Cancer
CCA	Cholangiocarcinoma
PAs	Pleomorphic Adenomas
PanIN1	Pancreatic Intraepithelial Neoplasia 1
DBD	DNA-Binding Domain
NLS	Nuclear Localization Signal
NES	Nuclear Export Signal
TAD	Trans-Activation Domain
PI3K/AKT/mTOR	Phosphatidylinositol 3-Kinase/Protein Kinase B/Mammalian Target of Rapamycin
JAK/STAT3	Janus Kinase/Signal Transducer and Activator of Transcription 3
JNK	c-Jun N-Terminal Kinase
AMPK	AMP-Activated Protein Kinase
ERK	Extracellular Signal-Regulated Kinase
IKKβ	Inhibitor of Nuclear Factor Kappa B Kinase Subunit Beta
Atg7	Autophagy Related 7
G6PC	Glucose-6-Phosphatase Catalytic Subunit
IL-6	Interleukin 6
MMP9	Matrix Metallopeptidase 9
CYP7A1	Cytochrome P450 Family 7 Subfamily A Member 1
PKCζ	Protein Kinase C Zeta
STK3	Serine/Threonine Kinase 3
TP53INP1	Tumor Protein P53 Inducible Nuclear Protein 1
CDKN1A/p21	Cyclin-Dependent Kinase Inhibitor 1A
CCL20	C-C Motif Chemokine Ligand 20
CSF-1	Colony-Stimulating Factor 1
MDM2	Murine Double Minute 2
SKP2	S-Phase Kinase-Associated Protein 2
CTSL	Cathepsin L
5-FU	5-Fluorouracil
Smad2/3	Drosophila Mothers Against Decapentaplegic Protein 2/3
SIRT1	Silent Information Regulator 1
VEGF	Vascular Endothelial Growth Factor
OS	Overall Survival
DIM	3,3’-Diindolylmethane
APC2	Adenomatous Polyposis Coli 2
FasL	Fas Ligand
Bim	Bcl-2-Interacting Mediator of Cell Death
EMT	Epithelial–Mesenchymal Transition
Tregs	Regulatory T Cells
Th17	Helper T Cell 17
CAR-T cells	Chimeric Antigen Receptor T Cells
